# Defining early health technology assessment: building consensus using Delphi technique

**DOI:** 10.1017/S0266462325100123

**Published:** 2025-06-02

**Authors:** Janneke P.C. Grutters, Janet Bouttell, Payam Abrishami, Sulafa. Y.M. Ahmed, Amanda Cole, Dalia Dawoud, Carla Fernández-Barceló, Geert W.J. Frederix, Neil Hawkins, Jonathan Karnon, Sharon Leadbitter, Christopher McCabe, Jani Mueller, Samuel Owusu Achiaw, Andrew Partington, Laura Sampietro-Colom, Abril Seyahian, Rabia Sucu, Michelle Tew, Sasha van Katwyk, Yves Verboven, Yi Wang, A Working Group of HTAi

**Affiliations:** 1 Science Department IQ Health, Radboud University Medical Centre, Nijmegen, The Netherlands; 2 Centre for Healthcare Equipment and Technology Adoption, Nottingham University Hospitals NHS Trust, Nottingham, UK; 3 Health Economics and Health Technology Assessment, School of Health and Wellbeing, University of Glasgow, Glasgow, UK; 4 VDL Enabling Technologies Group, Eindhoven, The Netherlands; 5 Faculty of Engineering and Technology, University of Gezira, Wad Madani, Sudan; 6 Office of Health Economics, London, UK.; 7 Faculty of Pharmacy, Cairo University, Cairo, Egypt; 8 National Institute for Health and Care Excellence (NICE), London, UK; 9 Coreva Scientific GmbH & Co. KG, Königswinter, Germany; 10 Department of Epidemiology and Health Economics, Julius Center for Health Sciences and Primary Care, University Medical Center Utrecht, Utrecht University, Utrecht, The Netherlands; 11 HAN University of Applied Sciences, Nijmegen, The Netherlands; 12 Flinders Health and Medical Research Institute, College of Medicine and Public Health, Flinders University, Adelaide, Australia; 13 TACS Healthcare, Sydney, Australia; 14 Centre for Public Health and Queen’s Business School, Queens University Belfast, Northern Ireland; 15 Department of Public Health, University of Pretoria, Hatfield, South Africa; 16 Centre for Healthcare Resilience and Implementation Science, Australian Institute of Health Innovation, Macquarie University, Sydney, Australia; 17 Assessment of Innovations Unit, Clinic Barcelona University Hospital, Barcelona, Spain; 18 Management Sciences for Health, Arlington, VA, USA; 19 Melbourne Health Technology and Value Assessment Collaboration, Melbourne Health Economics, Melbourne School of Population and Global Health, The University of Melbourne, Melbourne, Australia; 20 Institute of Health Economics, Edmonton, AB, Canada; 21 EU4HealthSolution, Brussels, Belgium; 22 Saw Swee Hock School of Public Health, National University of Singapore and National University Health System, Singapore

**Keywords:** technology assessment, biomedical, terminology as a topic, value-based health care, decision making, translational research

## Abstract

Although early health technology assessment (HTA) is increasingly being used to guide and inform decisions on product development, a consensus definition is currently lacking. A working group under the HTA International Society was established to develop a consensus-based definition of early HTA. The working group developed a definition using an iterative process that comprised five stages of work and included a two-round Delphi survey with 133 respondents in the first and 99 respondents in the second round of the survey, with various backgrounds and levels of expertise. Following this process, the working group reached the first consensus-based definition of early HTA, which is an HTA conducted to inform decisions about subsequent development, research, and/or investment by explicitly evaluating the potential value of a conceptual or actual health technology. In total, 86 (87 percent) of the 99 panelists who participated in the second round of the Delphi survey either strongly agreed or agreed with this definition. This consensus definition represents an important milestone in early HTA. It will enhance the uniformity of terminology, increasing the visibility of research and policy in this field. We also hope that it will act as a catalyst sparkling further research and developments in this discipline.

## Introduction

According to the glossary definition (1), health technology assessment (HTA) is a multidisciplinary process that uses explicit methods to determine the value of a health technology at different points in its life cycle. The most familiar form of HTA is work conducted by HTA agencies, on behalf of healthcare systems or other payers, to inform reimbursement or adoption decisions (including price negotiations). HTA is often used to inform decisions about the adoption, use, or pricing of pharmaceuticals, medical devices, and other technologies (defined widely in the HTA glossary definition) (2). However, HTA is also performed in earlier stages of the development of a technology to inform premarket decisions (3). This has been named “early HTA” or “development-focused HTA” and encompasses a broad range of work and technologies (3–7). For example, early HTA could inform private or public innovators or investors during research into a new pharmaceutical, medical device, or diagnostic; innovators looking to improve hospital processes; or potential users in the early stages of development, looking into the design or alternative adoption strategies of an innovative health technology (5;8). Early HTA can be conducted within healthcare settings as part of a broader hospital-based HTA (9), for example, to help inform the in-house development of technologies, but is also performed within life science industries that supply technologies into the health system (10–13).

Early HTA is increasingly being used in all research and development phases, at different technology readiness levels. It has great potential to reduce research waste, ensuring that investment goes to technologies that are expected to create value, and are optimized to ensure they are fit for purpose (14–17). This focus on early assessment is in line with multiple policy initiatives from health systems and other payers set to provide information earlier in the life cycle on the potential value of a new technology to guide investment and assessment prioritization (18–21). Horizon scanning is the systematic identification of health technologies that are new, emerging, or becoming obsolete and that have the potential to affect health, health services, and/or society (22). Early HTA, on the other hand, refers to the assessment of these new and emerging technologies.

Often, stakeholders utilizing early HTA do not explicitly state that they consider their activity to fall within this remit and use other terms to describe it. For example, pharmaceutical companies use a range of “target assessment” frameworks in which important activities include the defining of unmet need and clinical differentiation (23). Both activities form part of early HTA, often as a first step. Much early HTA, particularly that undertaken primarily to inform innovators, remains unpublished as it may be commercially sensitive (24). Early HTA draws on a suite of complementary methods to assess the need for the innovation or develop target product profiles, such as interviews, expert (stakeholder) elicitation, and health economic modeling (3;4;7;17;25). These methods can be used to explore the potential value of a technology in development using scenarios based on real-world settings, for example, reflecting alternative positions in a clinical pathway a technology could be used in, or considering alternative implementation contexts and the interoperability of a technology with existing health systems.

With increasing use of early HTA, there has been considerable debate over its precise definition, and if and how it differs from related concepts such as “early awareness,” “early dialogue,” “early (scientific) advice,” and “development-focused HTA.” Considering the multiple policy initiatives emerging in different parts of the world (18–21) and the heterogeneity in the field, clear guidance on terminology, methods, and reporting of early HTA would greatly assist practitioners, as well as journals seeking to ensure the quality of published work. The purpose of this study is to address the first of these issues and establish consistency in terminology. A working group under the HTA International Society (HTAi) was initiated to establish consensus on the definition of early HTA. This article reports the findings of this group and presents the first consensus-based definition of early HTA.

## Methods and results

**Figure 1.**
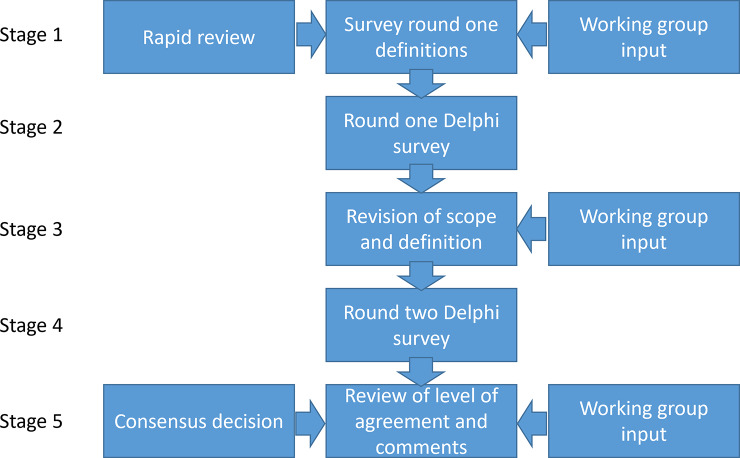
Stages of the Delphi process.

Our study used an iterative process comprising five stages of work undertaken by two bodies: the working group established under the auspices of HTAi and the panel who responded to the two stages of the consensus Delphi process. We chose to undertake a Delphi process as it is an appropriate method to reach consensus (26). With the Delphi process, we wanted to reach as many people working in the field as possible. We report the methods and results of these stages chronologically, in line with the Guidance on Conducting and Reporting Delphi Studies (CREDES) (26) (see Figure 1). The working group was established from a group of individuals interested in early HTA who were brought together by the first authors (JG and JB) following a call across their networks and to attendees of the HTAi Annual Meeting in the Netherlands in June 2022. Members of this wider group volunteered to join a terminology working group. Further members were added when it was formally accepted as a working group of HTAi in the summer of 2023. An advisory board was set up with five experts from different backgrounds. The total working group consisted of 17 core working group members and 5 advisory board members. These 22 people are referred to as the working group. The panel for the Delphi survey comprised all those who responded to the first round of the survey. The characteristics of both the working group and the panelists can be found in the Supplementary Materials.

### Stage 1: development of definitions and supporting materials

Stage 1 started with a rapid review of reviews of early HTA, undertaken by JG in February 2023 based on an update of the search set out in Grutters et al. (4). Keywords were “(‘early health technology assessment’ OR ‘early evaluation’ OR ‘early assessment’) AND ‘methodology’ AND ‘review’.” The search added 85 articles to the previous review, of which 2 were considered relevant. Working group members were invited to add relevant review articles. A total of 11 articles were identified, including 9 separate definitions (3;5;6;8;24;27–32). These were set out in the materials circulated in the first round of the Delphi survey (see Supplementary Materials). JG and JB developed an initial suggested definition based on the output from this review, as well as the HTA glossary definition of HTA (1). These materials were then forwarded to the working group for their consideration. The working group decided to define the terms “early HTA,” “development-focused HTA,” and “early dialogue.” Other terms were used in the articles reviewed, but the working group preferred early HTA due to its prominence in the literature and development-focused HTA, as it captured the distinct nature of work undertaken to inform the development of health technologies. Early dialogue was included because when setting up the working group, there was much discussion about whether and how early HTA was different from early dialogue. Often used terms such as early economic evaluation or early health economic modeling (28;31;32) were considered specific methods that could be used to inform early or development-focused HTA and were, therefore, not included in the scope of the study. The initial definition of early dialogue was taken from a recent publication by Blankart et al. (33). To avoid bias, the first survey included a question asking panelists whether they agreed with the use of the terms we had suggested and asking for their own suggestions.

The first survey comprised background questions, definitions of “early HTA,” “development-focused HTA,” and “early dialogue”; a table setting out a detailed definition of early HTA in stages; and a table reconciling the suggested definition of early HTA with the nine definitions found in the rapid review. The full text circulated was refined iteratively through consultation with the working group. The final draft of the survey was piloted with five colleagues who were not involved with this work, and no changes to the context or structure were required. The survey, including the initial definitions, is included in the Supplementary Materials. The Supplementary Materials also include the characteristics of the working group and panelists who responded to the survey. A total of 13 members of the working group were panelists in Rounds 1 and 2. Their characteristics are included in all relevant columns.

### Stage 2: Round 1 Delphi survey

A protocol for the Delphi study was developed by the working group (see Supplementary Materials). An ethical waiver was received from Radboud University Medical Center as no patients were included, and participation in the study was not associated with any risks or harms. The first round of the Delphi survey was circulated on 26 October 2023, with responses required by 24 November 2023. The survey was accompanied by an information sheet for participants (see Supplementary Materials). The survey started with an explicit consent statement that the respondents were asked to agree with. The purpose of the first round of the survey was to elicit qualitative comments rather than to seek consensus. We sought to reach a wide range of stakeholders with an interest in early HTA, including health policy makers and those in academia, HTA agencies, consultancy, and industry. As early HTA is an emerging field, we sought to be inclusive of all interested individuals regardless of their level of experience. We distributed the survey link through personal networks and social media and encouraged panelists to forward the survey link on to interested parties in their own networks. HTAi also distributed the invitation to all Interest Groups within their organisation and via their newsletter. Panelists in Round 1 were asked to provide their email address if they wished to be included in Round 2.

We received 133 responses to Round 1 of the survey; all of them gave informed consent. A total of 119 (of 133) panelists included their email addresses to be invited to participate in Round 2, and 114 panelists included free text comments for consideration.

In response to the question about whether panelists agreed with the use of the three terms we sought to define: “early HTA,” “development-focused HTA,” and “early dialogue,” many panelists found it difficult to distinguish between “early HTA” and “development-focused HTA.” Panelists felt that the concept of development-focused HTA covered the earliest stages of early HTA and that - if included - another complementary term covering the later stages of early HTA should also be included. There were contrasting views about the definition of “early dialogue” and they fit with early HTA. Multiple panelists regarded early dialogue as a method of stakeholder involvement used for early HTA. If it was a specific type of stakeholder involvement, as the proposed definition suggested, panelists suggested changing the term accordingly, for example, “early regulatory dialogue.”

In response to our question about any other suggested terms, panelists proposed 26 alternative terms, with 1 term, “developmental HTA,” suggested twice.

### Stage 3: decision on scope and revision of definition

Stage 3 involved collaborative consideration by the working group of the feedback received from Round 1 of the survey and amendment of the definition of early HTA. At this stage, we also discussed the characteristics of the panel and identified some additional questions regarding the panelists’ characteristics that we wished to ask in Round 2. Given the responses on development-focused HTA being a subset of early HTA, the working group considered that introducing another complementary term covering the later stages of early HTA would create confusion. Hence, the working group decided to drop development-focused HTA and concentrate on the definition of early HTA, as the more comprehensive and recognized term. The working group also decided that, given the multiple policy initiatives in the area of early dialogue at present and the greater expertise elsewhere in HTAi on this topic, we would not seek to develop a consensus definition for this term. Regarding the alternative terms that were suggested by the panelists, many were suggested as mirror terms for development-focused HTA or to provide two terms to subdivide early HTA. In view of the absence of a dominant alternative, the working group decided to focus only on the definition of the single term “early HTA.”

The working group reviewed the responses, and the main themes were discussed at length. An iterative amendment process was undertaken, comprising a meeting of the working group and subsequent group emails, until the working group was satisfied that the definition reflected their understanding of early HTA. At this stage, we also consulted a panelist and a lexicographer who were involved in the HTA Glossary. Box 1 sets out the initial definition circulated with Round 1 and the final definition arrived at by the working group. Based on the advice offered, “HTA” appears as the first phrase in the definition to link directly to the overall definition in the HTA glossary. Early HTA is a subset of HTA, which means that concepts from the main definition, such as “to promote an equitable, efficient, and high-quality health system” are implied and, therefore, not required in our core definition. In the Supplementary Materials, we explain the working group’s responses to feedback received in the first round and how that was considered in the different aspects of the definition.

Box 1. Definition of early HTA included in the two rounds of the survey

**Definition included in Round 1 of the Delphi survey**

Early HTA is a formal, systematic, transparent, and multidisciplinary process that uses explicit methods, both quantitative and qualitative, to explore the potential and/or expected value of a health technology*, including the associated uncertainty, before or alongside the technology development process. Stages at which early HTA can be undertaken include the concept/discovery stage, prototype/proof of concept stage, and research/evidence development stage. The stages have an impact upon the evidence/data available, the questions to be answered, the methods to be used, and the audience for the work. The purpose is to provide innovators with insight about the potential value** for the health system and commercial viability of a technology, and to inform decision making about the (clinical) need, design of a technology, positioning of the technology in the care pathway, further research needed to prove value, and potential for future market access and adoption, to promote a high-quality health system.

* A health technology is an intervention developed to prevent, diagnose, or treat medical conditions; promote health; provide rehabilitation; or organize healthcare delivery. The intervention can be a test, device, medicine, vaccine, procedure, program, or system (definition from the HTA Glossary; http://htaglossary.net/health+technology).

**The dimensions of value for a health technology may be assessed by examining the potential intended and unintended consequences of using a health technology compared to existing alternatives. These dimensions often include clinical effectiveness, safety, costs, and economic implications; ethical, social, cultural, and legal issues; organizational and environmental aspects, as well as wider implications for the patient, relatives, caregivers, and the population. The overall value may vary depending on the perspective taken, the stakeholders involved, and the decision context

**Accepted consensus definition**

Early health technology assessment

A health technology assessment (HTA) conducted to inform decisions about subsequent development, research, and/or investment by explicitly evaluating the potential value^1^ of a conceptual or actual health technology^2^.

1The dimensions of value for a health technology may be evaluated by examining the intended and unintended consequences of using a health technology compared to existing alternatives. These dimensions often include clinical effectiveness, safety, costs, and economic implications; ethical, social, cultural, and legal issues; organizational and environmental aspects, as well as wider implications, for example, for the patient, relatives, caregivers, innovators, and the population. The overall value may vary depending on the perspective taken, the stakeholders involved, and the decision context.2An intervention developed to prevent, diagnose, or treat medical conditions; promote health; provide rehabilitation; or organize healthcare delivery. The intervention can be a test, device, medicine, vaccine, procedure, program, or system.

HTA, health technology assessment.

### Stage 4: Round 2 Delphi survey

In the second round of the survey, we asked participants to respond on a Likert scale to indicate whether they strongly agreed, agreed, were neutral, disagreed, or strongly disagreed with the definition. They were asked to provide comments to support their responses. Participants who had provided their email addresses in Round 1 were sent a personal link to complete Round 2. As prespecified in the protocol, we considered consensus reached if 70 percent of panelists or more either strongly agreed or agreed with the definition. Panelists were asked to provide their name and affiliations if they wished to be acknowledged in this article. In addition, we asked them questions about their geographical background and follow-up questions about their expertise in (early) HTA.

**Figure 2.**
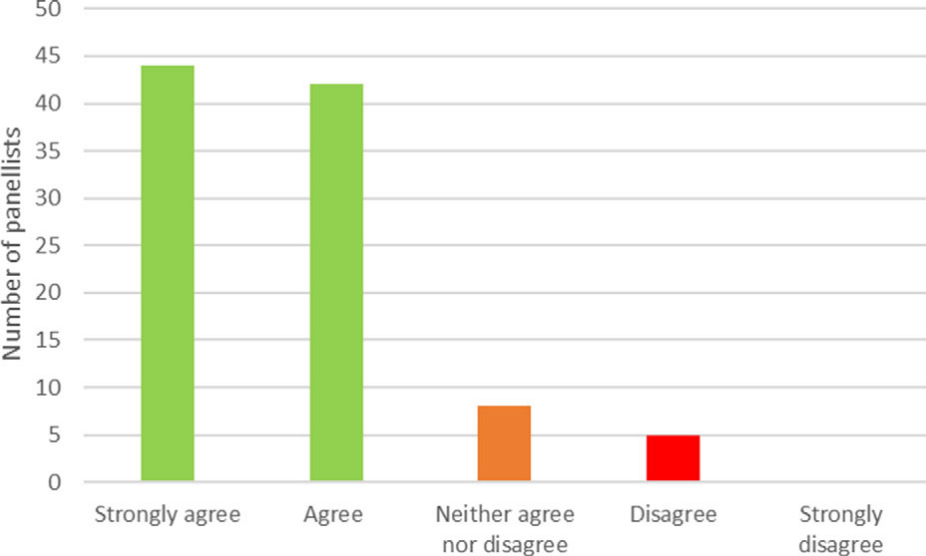
Level of consensus on the provided definition of early HTA. HTA, health technology assessment.

Of the 119 panelists who provided their email addresses in the first round, 99 (83 percent) took part in the second round. Figure 2 shows the level of agreement reached in the second round. In total, 86 (87 percent) panelists either strongly agreed or agreed with the definition. This compares with a consensus threshold of 70 percent set in our protocol. Eight panelists (eight percent) neither agreed nor disagreed, and five (five percent) disagreed. Of the 13 members of the working group who were also panelists, 5 agreed and 8 strongly agreed with the definition. Excluding these 13 individuals results in a level of agreement of 85 percent, with 73 panelists agreeing or strongly agreeing from a total of 86. Excluding panelists with no experience of either early HTA or early dialogue, the level of consensus is 88 percent.

### Stage 5: decision on consensus

In the fifth and final stage of the study, the working group considered the responses to the second round and decided whether any further amendment was required. The working group unanimously concluded that, given the strong level of agreement, the second definition, set out in Box 1, would be adopted as the consensus definition. Free text comments from the second round of the survey focused on three key areas: what early HTA can and cannot do; confusion with or between early dialogue/early awareness/early scientific advice, and the timing of early HTA.

#### What early HTA can and cannot do

One panelist commented that early HTA cannot include early ethical, social, cultural, legal, organizational, and environmental aspects. The working group felt that early HTA can consider these elements and that it was important to emphasize the relevance of exploring these aspects at an early stage of development to anticipate later issues, even though this is currently not often included in early HTA (34). Another panelist expressed concern that early HTA would be “inaccurate” due to a lack of detail and a fast-moving environment. The working group felt that this comment misunderstood the purpose of early HTA. Given the purpose of early HTA is to inform decisions about subsequent development, research, and/or investment, an early HTA would highlight a fast-moving therapeutic or competitive environment and incorporate this uncertainty into analyses. Although it could be argued that all HTA is, on some level, imprecise, economic evaluation as part of early HTA does not typically give a definitive answer to a binary question about whether a health technology is cost-effective or not. Rather, it is intended to identify the key parameters that will influence cost-effectiveness and provide some guidance about threshold levels of performance that may be required for a technology to add value. Understanding the needs of stakeholders for a technology and the conditions under which it can provide value for money is particularly important in fast-moving therapeutic and competitive environments.

#### Confusion between early dialogue/early awareness/early scientific advice

The responses highlighted some confusion on how early HTA relates to early dialogue, early awareness, and early scientific advice. Possibly, this is because early HTA undertaken within companies or on innovators’ behalf by consultants and academics is largely unseen. Panelists from HTA agencies are aware that their own or associated agencies’ horizon scan for emerging technologies (early awareness) and engage with innovators to discuss process and evidence requirements (early scientific advice). They may be less aware of early HTA, which occurs at a much earlier stage of development than these activities and is not readily visible to them. Since early dialogue explicitly concerns the interaction between the innovator and HTA agency and/or regulatory body, it is different from the method of stakeholder involvement that can be used as a qualitative method for performing an early HTA, which generally includes a broader set of stakeholders. Table 1 gives an illustration of the working group’s view of how these activities relate to early HTA.

**Table 1.** Relationship between early HTA, early awareness, and early dialogue/scientific advice

	Early HTA	Early dialogue/ early scientific advice	Early awareness /horizon scanning
Stakeholders involved	Innovators Funders Healthcare providers Clinicians Patients and their advocate groups Technology transfer offices	Innovators Regulators HTA agencies	Regulators HTA agencies Healthcare providers
Purpose	To inform the development of a technology, position in the clinical pathway, and value proposition	To ensure that innovators are aware of the evidence requirements of regulators and HTA agencies (33)	To identify new and emerging technologies and assess their potential impact on health, health services, and/or society (22)
Timing	TRL < 8 Preconcept stage to research and evidence generation stage	TRL = 5–8 Prototype and proof of concept (small-scale pilot testing) to research and evidence generation (large-scale testing)	TRL = 5–9 Prototype and proof-of-concept (small-scale pilot testing) to adoption and implementation (market access, adoption, and post-market surveillance)
Conducted/commissioned by	Innovators Funders Healthcare providers	Innovators	Regulators and/or HTA agencies Healthcare providers

HTA, health technology assessment; TRL, technology readiness levels

#### Timing of early HTA

Some panelists felt it was important to specify in the definition at what stage an HTA is “early,” in contrast to “not early” HTA. The working group felt that the only clear distinction between early and other forms of HTA relates to the decision problems that the respective assessments are intended to inform. The second definition (Box 1) is structured to place early HTA as a subset of HTA with a clear purpose that is different from, for example, HTA performed to inform reimbursement decisions. Further details provided in Table 2 illustrate the typical timing of early HTA. Panelists felt it would be useful to be explicit about several aspects of early HTA, such as who requests, carries out, and pays for the HTA; what the outputs are; whether the process is confidential; and the role of the HTA agency. The working group acknowledged the relevance of these questions, but noted that the answers will vary. For example, early HTA activities can be performed by a consultancy company to inform an innovator about the potential value for money of their technology or idea, in which case it will be paid for by the innovator, and the process is probably confidential. However, early HTA could be facilitated by an academic expert, paid for by a public research funder, to inform decisions on funding a clinical study on a new technology. We added details covering these points to Tables 1 and 2. Although the aim of the Delphi survey was to adopt a broad definition of technology and to make the definition of early HTA technology agnostic, we acknowledge that some technologies, such as orphan drugs, digital health, or service innovations, may deviate from standard health technologies, and our general descriptions may not capture every nuance.

**Table 2.** Additional detail by stage of technology development

HTA terminology	HTA			
	Early HTA		Transitioning from early HTA	
	Stage 1: concept and discovery	Stage 2: prototype and proof of concept	Stage 3: research and evidence generation	Adoption and implementation
Technology-specific evidence	Bench, in silico and animal studies, formulation, pharmacokinetic absorption, distribution, metabolism, and excretion (ADME) studies, establish safety, and user studies of an early prototype. Often, no efficacy or effectiveness evidence available.	Evidence of safety and efficacy from a small sample Usability/patient acceptance studies Preclinical studies, including good laboratory practice, animal safety, and toxicity. Phase 1 and Phase 2a clinical trials were conducted.	Safety and clinical effectiveness study. May include randomized controlled trial or observational evidence, depending on regulatory requirements. Phase 2b and Phase 3 clinical trials were conducted.	Evidence of safety, clinical effectiveness, quality of life, and cost implications is available but may be limited to certain settings, populations, or jurisdictions. Post-market /real-world studies.
Typical project scenario	Technology-driven: either an emerging and generalized technology with broad application across several potential indications, or a technology with specific features requiring a target indication, setting, and position in a pathway. Needs-driven: no technology, yet specified, with emphasis on identifying and designing features required to realize a patient, payer, or innovator improvement.	Potential indications and/or features have been narrowed down. Exploration of position in the pathway and setting.	Technology and market development may continue.	Regulatory evidence base available. The indication is clear from the regulation. The reimbursement application is in process or completed.
Potential stakeholders	Innovators (industry/academic/health care professional). Funders (private or public entities; funding research, evidence generation, and/or technology development). Healthcare providers. Clinicians. Patients and their advocate groups. Technology transfer offices.	Innovators (industry/academic/health care professional). Funders (private or public entities; funding research, evidence generation, and/or technology development). Healthcare providers. Clinicians. Patients and their advocate groups. Technology transfer offices.	Innovators (industry/academic/health care professional). Funders (private or public entities; funding research, evidence generation, and/or technology development). Healthcare providers. Clinicians. Patients and their advocate groups. Technology transfer offices. Regulators.	Innovators (industry/academic/health care professional). Funders (private or public entities; funding research, evidence generation, and/or technology development). Healthcare providers. Clinicians. Patients and their advocate groups. Technology transfer offices. Regulators.
Example of appropriate methods (not comprehensive or prescriptive)	Qualitative methods: Care pathway analysis Stakeholder engagement (e.g., interviews/focus groups/surveys with range of stakeholders) Health economic modeling: Using data from literature, preclinical data, and assumptions Simple exploratory models Use of headroom and threshold estimates Exploration of structural uncertainty using scenarios	Qualitative methods: Care pathway analysis Stakeholder engagement (e.g., interviews/focus groups/surveys with range of stakeholders) Health economic modeling: Using early data from small studies, data from literature, and assumptions Simple exploratory models Use of headroom and threshold estimates Exploration of structural uncertainty using scenarios	Qualitative methods: Care pathway analysis Stakeholder engagement (e.g., interviews/focus groups/surveys with range of stakeholders) Health economic modeling: Using early data from larger studies Probabilistic models Value of information analysis Budget impact assessment	Qualitative methods: Care pathway analysis Stakeholder engagement (e.g., interviews/focus groups/surveys with range of stakeholders) Health economic modeling: Prepared in accordance with context-specific requirement (e.g., NICE reference case)
Key questions about the development of the technology	What characteristics does the technology need to deliver on the proposed value proposition claims? What evidence needs to be generated to meet future regulatory/HTA requirements? What is the feasibility of collecting evidence required to demonstrate value, assessment of epidemiology, natural history, and burden of disease?	Questions as per Stage 1 plus: How usable is the technology? Would this be acceptable to intended users? Should we invest in preliminary data collection to inform safety and effectiveness? What are the barriers/facilitators to adoption and/or implementation?	Questions as per Stage 2 plus: What logistical considerations are required to provide timely access? What are the implementation considerations (health system readiness, workforce planning, and resource allocations)? What are the timeframes to meet to ensure timely access?	
Key questions about the positioning of the technology	What is/are the current clinical pathway/s? How would the technology change the care pathway? What is the room for improvement? What is the targeted patient population?	Questions as Stage 1	Questions as Stage 2	
Key questions about the value proposition of the technology	Who are the main stakeholders for the future adoption of the technology? What is the potential impact on health, cost, resource availability, equity, accessibility, efficiency, or sustainability? What is the minimum level of outcomes that is needed, given the threshold costs? What is the maximum cost the expected outcomes could support, given the threshold costs? What evidence is required to demonstrate that the technology is likely to deliver value as defined by the chosen decision maker/s? How does the decision maker weigh the different elements of impact/value? What other technologies are on the horizon that may change the competitive or therapeutic environment? What is the market size?	Questions as per Stage 1 plus: At what price/performance characteristics is the technology likely to be cost-effective in selected jurisdictions? Are the expected revenues and commercial return on investment sufficient to develop the technology?	Questions as per Stage 2 plus: Does preliminary evidence on safety and effectiveness justify investment in large-scale testing?	Is the technology likely to be cost-effective in the specific population, position in the pathway and jurisdiction at a set price? Should we fund/cover/adopt the new technology? Should the conditions be placed on adoption for restricted coverage, risk-adjusted pricing, and further evidence generation? What are the financial implications and considerations to ensure continuing access?
Technology readiness levels (TRL) (35)	TRL < 4: review of scientific knowledge base, development of a technology’s hypothesis, identification, and characterization of candidate technologies, optimization, and initial demonstration of safety and efficacy.	TRL = 5–6: advanced characterization of technology and initiation of manufacturing, and/or staff recruitment. Regulated production, regulatory submission, and clinical data.	TRL = 7–8: scale-up, initiation of good manufacturing practice, process validation, and the Phase 2 clinical trials.	TRL = 9: market access, adoption, and the post-market surveillance.

HTA, health technology assessment; TRL, technology readiness levels.

### Additional details on the stages of early HTA

In the first round of the survey, we included a detailed table that delineated early HTA into three stages, shown alongside the phases of development of a technology (see Supplementary Materials). We amended the table in response to the feedback in Round 1 (Table 2). Specific changes include a recognition that, as the development of the technology proceeds into what we have termed “Stage 3: research and evidence generation,” HTA can still be early, but it does not necessarily have to be, depending on the purpose of the assessment. If the purpose of HTA activities at this stage is to inform risk sharing and ongoing monitoring arrangements as part of reimbursement/adoption decisions rather than to directly inform decisions to adapt or develop the technology, it is no longer deemed early. We also added details of typical methods at each stage of development, including some comments on how uncertainty may be explored, as this was a common request in the feedback to Round 1. It should be noted that these are examples only and not intended to be exhaustive or prescriptive. Table 2 should not be seen as part of the consensus definition, as it was not included in the second round. However, we felt it addressed most of the comments that were made on the definition in Round 2 of the Delphi process.

## Discussion

We undertook a five-stage process, including a two-round Delphi survey, which produced consensus on a definition of early HTA. Based on this process, early HTA is defined as “an HTA conducted to inform decisions about subsequent development, research, and/or investment by explicitly evaluating the potential value of a conceptual or actual health technology”. A total of 11 previous articles had suggested 9 definitions for early HTA or related terms (see Supplementary Materials) (3;5;6;8;24;27–32). Several of these definitions were limited to the health economic modeling component of early HTA (28;31;32), whereas our definition considers wider implications by incorporating the note from the HTA glossary definition of HTA on the dimensions of value, albeit slightly amended to include implications for the innovator. Pietzsch and Pate-Cornell (8) and Ijzerman and Steuten (6) both recognize that the purpose of early (health) technology assessment is to inform future development, with the former explicitly recognizing that investment and design decisions may be informed. Ijzerman et al. (3) explicitly recognize that industry may be the primary audience defining early HTA as “all methods used to inform industry and other stakeholders about the potential value of new medical products in development.” Fasterholdt et al. (27) defined early assessment as “being performed when the initial selection of ideas or rough prototyping has taken place, but before large-scale testing or traditional clinical research. Hence, early assessment is based on data from early phases, that is, feasibility, pilot, or initial effect data.” This focus on a specific stage in development or the un/availability of specific data is useful in the definition of early HTA, and we have included both aspects in our detailed table (Table 2); however, the working group felt that the distinctive feature of early HTA is that it is intended to inform decisions around development, research, and investment decisions. The availability or otherwise of data is not a defining characteristic of early HTA.

We present the first consensus-based definition of early HTA. The strengths of our approach are the extensive experience and different perspectives represented in our working group and by our Delphi panel members. We have representation from most geographic areas, although we acknowledge that there is a preponderance of involvement from Europe and Australia. Although there is no recognized standard for the conduct or reporting of consensus exercises such as ours, we have followed the CREDES best practice guidelines (26). We set out our methodology in our protocol, worked under the oversight of the Scientific Development and Capacity Building Committee of HTAi, and have reported all aspects of our process transparently. Our study has a number of limitations. Both the working group and the Delphi panel had a strong representation from Australia and Europe. Moreover, most of the working group members and panelists were from academia and mostly had experience with quantitative methods. This is not surprising, given that most early HTA activities focus on health economics and are performed in Europe and Australia. Both the working group and panel were open to anyone interested, and we did not have a predefined threshold for the representation of certain stakeholders, regions, or experience. Our panel and working group thereby seem to be a good representation of the current interest in and use of early HTA.

The development of this consensus definition of early HTA is important because it provides clarity and raises the profile of the field. Although it fits within the umbrella definition of HTA developed by an international joint task group co-led by the International Network of Agencies for Health Technology Assessment and HTAi (1), it does suggest an extension of the concept of “value” in that definition to include wider implications for innovators. It also makes clear that early HTA is not restricted to the activities of HTA agencies but involves a wide range of actors from the very earliest stages and may precede the development of the technology itself, with much work remaining unpublished and potentially “below the radar.” It clearly distinguishes early HTA from related but distinct activities of early awareness and early dialogue/early scientific advice. Developing a consensus definition of these terms was beyond the scope of this study, but it would further clarify the differences between the activities. We urge authors to identify their papers as early HTA, where appropriate, and use our detailed table (Table 2) to report the stage of development of the technology and the level of evidence available. We encourage journal editors to reinforce the use of this uniform terminology to improve visibility. Next steps for our group include the submission of the consensus definition to the HTA glossary and working on methods and reporting of early HTA. In developing methods, it will be useful to relate early HTA to other fields of research, such as bioethics, philosophy of technology, responsible research and innovation, and decision making under deep uncertainty. In addition, we stress that, like all definitions, this is a “living” definition that may need to be updated in time to reflect the evolution continuously happening within the field of HTA.

Early HTA is performed to inform decisions about development. It provides an opportunity to assess the potential value of innovation before significant funds are committed, thus guiding investment decisions. We also advocate the adoption of an early HTA approach in a proactive sense to identify and describe specific clinical needs and the technology features required to meet them. Furthermore, early HTA provides the opportunity to ensure that technology is optimally designed and positioned to deliver the most value to a diverse range of stakeholders, including the innovators themselves, whether they are working within the healthcare system or in the industry. Early HTA, like HTA as a whole, seeks to promote an “equitable, efficient, and high-quality health system.”

## Conclusion

In this article, we have reported a five-stage process, including a two-round Delphi survey that developed and reached consensus on a definition of early HTA, which is “an HTA conducted to inform decisions about subsequent development, research, and/or investment by explicitly evaluating the potential value of a conceptual or actual health technology.” By providing a consensus-driven definition of early HTA, we hope to enhance uniformity and harmonization of terminology. In addition, we hope to lay the foundation for more discussion, research, and method development in this important field.

## Supporting information

Grutters et al. supplementary material Grutters et al. supplementary material

## Supplementary material

The supplementary material for this article can be found at http://doi.org/10.1017/S0266462325100123.
