# Supplementary material for: Defining early health technology assessment: building consensus using Delphi technique
Source: Int J Technol Assess Health Care. 2025 Jun 2;41(1):e34. doi: 10.1017/S0266462325100123 (PMC12178750; doi:10.1017/S0266462325100123)
Supplement: Grutters et al. supplementary material [file S0266462325100123sup001.docx]

Defining early Health Technology Assessment: building consensus using Delphi technique

Early HTA terminology Working Group of HTAi

# Supplementary materials

1. Delphi panellists’ characteristics
2. First round Delphi survey
3. Second round Delphi survey
4. Information sheet for participants
5. Protocol
6. Working group’s response to round 1 feedback
7. Panellists completing both rounds one and two who agreed to be acknowledged

# SM1 – Delphi panellists’ characteristics

**Background of working group and panellists in rounds one and two of Delphi surveys**

|  | **Round 1 (N=133)** | **Round 2 (N=99)** | Working group (N=22) |
| --- | --- | --- | --- |
| **Age** |  |  |  |
| 18 - 29 years old | 10 (8%) | 8 (8%) | 2 (9%) |
| 30 - 44 years old | 49 (36%) | 37 (37%) | 9 (41%) |
| 45 - 59 years old | 62 (47%) | 45 (45%) | 10 (45%) |
| 60+ | 12 (9%) | 9 (9%) | 1 (5%) |
| **Stakeholder group*** |  |  |  |
| Academia | 62 (45%) | 48 (48%) | 11 (50%) |
| Consultancy | 18 (13%) | 12 (12%) | 4 (18%) |
| Hospital | 2 (1%) | 2 (2%) | 3 (14%) |
| HTA agency | 9 (6%) | 4 (4%) | 1 (5%) |
| Industry | 18 (13%) | 13 (13%) | 2 (9%) |
| Patient representative | 6 (4%) | 5 (5%) | - |
| Policy maker/payer | 11 (8%) | 9 (9%) | - |
| Provider | 3 (2%) | 3 (3%) | - |
| Other | 4 (3%) | 3 (3%) | 1 (5%) |
| **Region** |  |  |  |
| Africa | - | 5 (5%) | 2 (9%) |
| Asia | - | 8 (8%) | 1 (5%) |
| Australia | - | 23 (23%) | 4 (18%) |
| Europe | - | 46 (46%) | 13 (59%) |
| North America | - | 10 (10%) | 2 (9%) |
| South America | - | 7 (7%) | - |
| **Involvement in types of innovation**** |  |  |  |
| Medical devices | 85 (63%) | 65 (66%) | 15 (68%) |
| Pharmaceuticals | 68 (51%) | 51 (52%) | 12 (55%) |
| Service innovations | 67 (50%) | 48 (49%) | 13 (59%) |
| Other | 11 (8%) | 11 (11%) | 3 (14%) |
| **Experience in using or conducting traditional or mainstream HTA** |  |  |  |
| Yes, 0-5 years of experience | 35 (26%) | 24 (24%) | 8 (36%) |
| Yes, 5-10 years of experience | 26 (19%) | 23 (23%) | 4 (18%) |
| Yes, 10-20 years of experience | 44 (33%) | 28 (28%) | 3 (14%) |
| Yes, over 20 years of experience | 21 (15%) | 19 (19%) | 7 (32%) |
| No | 7 (5%) | 5 (5%) | - |
| **Experience in conducting early HTA** |  |  |  |
| Yes, 0-5 years of experience | 49 (36%) | 36 (36%) | 7 (32%) |
| Yes, 5-10 years of experience | 23 (17%) | 18 (18%) | 1 (5%) |
| Yes, 10-20 years of experience | 15 (11%) | 10 (10%) | 8 (36%) |
| Yes, over 20 years of experience | 4 (3%) | 3 (3%) | - |
| No | 42 (31%) | 32 (32%) | 6 (27%) |
| **Do you conduct 'standard' HTA to inform reimbursement, adoption or similar decisions?**** |  |  |  |
| Yes, on behalf of HTA agencies | - | 28 (28%) | 6 (27%) |
| Yes, on behalf of industry | - | 30 (30%) | 6 (27%) |
| Yes, on behalf of healthcare institutions (e.g. hospitals) | - | 32 (32%) | 6 (27%) |
| Yes, on behalf of national funding bodies | - | 29 (29%) | 10 (45%) |
| Yes, on behalf of research institutions | - | 4 (4%) | 4 (18%) |
| Other | - | 4 (4%) | - |
| No | - | 20 (20%) | 2 (9%) |
| **Do you conduct 'early' HTA?**** |  |  |  |
| Yes, on behalf of HTA agencies | - | 15 (15%) | 1 (5%) |
| Yes, on behalf of industry | - | 32 (32%) | 9 (41%) |
| Yes, on behalf of healthcare institutions (e.g. hospitals) | - | 30 (30%) | 6 (27%) |
| Yes, on behalf of national funding bodies | - | 17 (17%) | 4 (18%) |
| Yes, on behalf of research institutions | - | 4 (4%) | 6 (27%) |
| Other | - | 3 (3%) | 1 (5%) |
| No | - | 30 (30%) | 8 (36%) |

* Please note that panellists could select only one role, while many panellists have dual roles. The categories ‘HTA agency’ and ‘Hospital’ were not provided but were derived from answers of panellists who selected ‘other’ and wrote ‘HTA agency’ or ‘Hospital’. Other panellists working at HTA agencies or similar agencies selected the ‘policy maker’ or ‘payer’ category.

** Totals may exceed 100% as panellists could select all appropriate options.

**Delphi panellists’ additional characteristics**

|  | **Round 1 (N=133)** | **Round 2 (N=99)** | **Working group (N=22)** |
| --- | --- | --- | --- |
| **Level of country development focus of work** |  |  |  |
| Low income countries | - | 17 (17%) | 6 (27%) |
| Middle income countries | - | 29 (29%) | 7 (32%) |
| High income countries | - | 80 (81%) | 19 (86%) |
| **Utilization of methodologies** |  |  |  |
| Qualitative methods | 2 (1%) | 2 (2%) | - |
| Quantitative methods | 45 (33%) | 30 (30%) | 4 (18%) |
| Both | 81 (60%) | 62 (63%) | 17 (77%) |
| Neither | 1 (1%) | 1 (1%) | - |
| Deliberative | 1 (1%) | 1 (1%) | 1 (5%) |
| Combine quantitative and qualitative evidence | 1 (1%) | 1 (1%) | - |
| Information retrieval for health technology resources | 1 (1%) | 1 (1%) | - |
| **Early HTA use for evidence synthesis/inform decisions** |  |  |  |
| Yes, 0-5 years of experience | 53 (39%) | 37 (37%) | 6 (27%) |
| Yes, 5-10 years of experience | 17 (12%) | 13 (13%) | 1 (5%) |
| Yes, 10-20 years of experience | 14 (10%) | 9 (9%) | 6 (27%) |
| Yes, over 20 years of experience | 4 (3%) | 4 (4%) | - |
| No | 45 (33%) | 36 (36%) | 9 (41%) |
| **Experience in early dialogue** |  |  |  |
| Yes, 0-5 years of experience | 40 (30%) | 29 (29%) | 6 (27%) |
| Yes, 5-10 years of experience | 17 (12%) | 14 (14%) | 2 (9%) |
| Yes, 10-20 years of experience | 9 (6%) | 5 (5%) | 3 (14%) |
| Yes, over 20 years of experience | 4 (3%) | 4 (4%) | - |
| No | 63 (47%) | 47 (47%) | 11 (50%) |

# SM2 – First round Delphi survey

Thank you for agreeing to participate in this study, conducted and coordinated by the Health Technology Assessment international (HTAi) Working Group on early HTA, led by Janet Bouttell (Nottingham University Hospitals Trust, UK) and Janneke Grutters (Radboudumc, the Netherlands). In this study we aim to develop a clear definition of the term “early HTA”. Such a definition is currently lacking and there is confusion with related terms such as “early dialogue” and “development-focused HTA”. Reaching consensus on what is meant by each of these terms will form a foundation for discussion and dialogue about the best methods to adopt and improve the profile of the field. You are part of a cohort of global experts who will be taking part in this study and influencing the output of this research.

This exercise is expected to take approximately 30 minutes of your time. Once you click 'Next', you will be directed to the informed consent page, followed by the proposed definitions, background information on the terminology, some background questions, and, finally, the Delphi exercise where you can provide your feedback. We will provide you with clear instructions on how to participate in this exercise.

Please note that you must finish the questionnaire for us to see your answers. If you wish to take part in the next round of the Delphi process, please provide us with your e-mail address (in the background questions). Your e-mail address will only be used for the invitation for the second round, your responses will be handled anonymously. If you participate in all rounds of the Delphi process, you will be given the opportunity to be acknowledged in the peer-reviewed article that will result from this work. In round 2 you will be asked if you want to be acknowledged, or prefer not to be acknowledged.

The deadline for submitting your answers is November 24. If you have any questions regarding this Delphi survey, you can contact Janet Bouttell ([Janet.Bouttell@nuh.nhs.uk](mailto:Janet.Bouttell@nuh.nhs.uk) ) or Janneke Grutters ([janneke.grutters@radboudumc.nl](mailto:janneke.grutters@radboudumc.nl)), at any point in the process.

There are 21 questions in this survey.

# Informed consent

**I agree to take part in this study.**

**I understand that participation in this study is voluntary and that I can withdraw from the study at any time.**

**I confirm that I have read and understood the information sheet provided for this study.**

**I understand that my data will be used in an anonymised format and my name will not be used in any reports or publications resulting from the study, unless I specifically consent to this.**

**I understand the meaning of the above statements.**

**I agree with these statements. ***

Please choose **only one** of the following:

- Yes
- No

If you agree with the statements, please continue to the next parts of the survey. If you do not agree with the statements, you cannot participate in the study. Please close this window.

## Proposed definitions

First, we will provide you with the proposed definitions. You will be asked to provide your feedback later.

**Early HTA**

Early HTA is a formal, systematic, transparent and multidisciplinary process that uses explicit methods, both quantitative and qualitative, to explore the potential and/or expected value of a health technology*, including the associated uncertainty, before or alongside the technology development process. Stages at which early HTA can be undertaken include the concept/discovery stage, prototype/proof of concept stage and research/evidence development stage. The stages impact upon the evidence/data available, the questions to be answered, methods to be used and the audience for the work. The purpose is to provide innovators with insight about the potential value for the health system and commercial viability of a technology, and to inform decision-making about the (clinical) need, design of a technology, positioning of the technology in the care pathway, further research needed to prove value and potential for future market access and adoption, in order to promote a high-quality health system.

* A health technology is an intervention developed to prevent, diagnose or treat medical conditions; promote health; provide rehabilitation; or organize healthcare delivery. The intervention can be a test, device, medicine, vaccine, procedure, program, or system (definition from the HTA Glossary; http://htaglossary.net/health+technology). The dimensions of value for a health technology may be assessed by examining the potential intended and unintended consequences of using a health technology compared to existing alternatives. These dimensions often include clinical effectiveness, safety, costs and economic implications, ethical, social, cultural and legal issues, organizational and environmental aspects, as well as wider implications for the patient, relatives, caregivers, and the population. The overall value may vary depending on the perspective taken, the stakeholders involved, and the decision context.

**Development-focused HTA**

Development-focused HTA is a specific type of early HTA that is mainly undertaken in the first two stages of early HTA (concept/discovery stage and prototype/proof of concept stage).  Its purpose is to provide innovators with insight about the potential (commercial) viability and design of a technology and the potential for future market access. It may also provide information for decision-makers about the potential of a technology in development to contribute to a high-quality health system.

**Early dialogue**

Early dialogue is the exchange between manufacturers and public institutions to obtain guidance on the evidence requirements for regulatory and reimbursement purposes. The purpose is to facilitate the flow of information between manufacturers, competent authorities (CA), and HTA agencies. Also known as early advice or (early) scientific advice.

**Detailed definition of Early HTA with stages**

|  | **Early HTA** | | | **Mainstream HTA**  **Implementation stage** |
| --- | --- | --- | --- | --- |
|  | **Stage 1 – concept/discovery stage** | **Stage 2 – prototype/proof of concept stage** | **Stage 3 – research and evidence generation stage** |  |
| **Technology Readiness Levels (TRL)**  **(Based on Seva et al, 2023)** | Up to and including TRL 4 from needs assessment, concept, development of a working prototype, including the observation and reporting of basic principles, development of an early prototype, bench and animal testing.  Proof of concept established. | TRL 5-6 – small scale pilot and feasibility testing. | TRL 7-8 – large-scale testing leading to regulatory approval. | TRL 9 – market access, adoption and post-market surveillance. |
| **Typical technology-specific evidence level** | No effectiveness evidence specific to the technology available.  Available technology-specific evidence may include:  Bench, in silico and animal studies, formulation, pharmacokinetic, absorption, distribution, metabolism and excretion (ADME) studies.  User studies of an early prototype. | Evidence of safety and effectiveness from small sample.  Usability/patient acceptance studies.  Pre-clinical studies, including Good Laboratory Practice, animal safety and toxicity.  Phase 1 and Phase 2a clinical trials conducted. | Effectiveness study.  May be randomised controlled trial or observational evidence depending on regulatory requirements.  Phase 2b and Phase 3 clinical trials conducted. | Regulatory evidence base available.  Evidence of clinical effectiveness, quality of life and cost implications (clinical utility) may be limited to certain settings or populations.  Post-market / Real-World studies. |
| **Typical project vignette** | **Technology-driven** - either an emerging and generalised technology with broad application across several potential indications, or a technology with specific features requiring a target indication, setting and position in a pathway.  **Needs-driven** - no technology yet specified, with emphasis on identifying and designing features required to realise a patient, payer or innovator improvement. | Potential indication and/or features have been narrowed down.  Position in pathway and setting may still be unclear. | Product and market development may be continuing.  Large scale testing.  Indication, position in pathway and setting should be clear. | Indication is clear from regulation. |
| **Audience** | Innovators/investors (industry/academic/clinician)  Funders or insurers  Technology transfer offices  Research funders  Patients and their advocate groups | Innovators/investors  Funder or insurer  Technology transfer offices  Health service users  Patients and their advocate groups | Innovators/investors  Funder or insurer  Technology transfer offices  Health service users  Patients and their advocate groups | Funders or insurers  Health service users  Patients and their advocate groups  Technology transfer offices |
| **Example questions to be answered** | What is/are the current pathway/s?  How would the technology change the care pathway?  What is the room for improvement?  What other technologies are on the horizon, which may change the competitive, therapeutic environment?  Do the potential or target claims/value proposition impact health, cost, access, equity, location of care, efficiency, sustainability?  What characteristics does the technology need to deliver on these claims?  What evidence is required to demonstrate that the technology is likely to deliver value as defined by the chosen decision-maker/s?  How does the decision maker weight the different elements of impact?  What is the minimum level of outcomes the expected costs could support, given threshold prices?  What is the maximum costs the expected outcomes could support, given threshold prices? Are the expected revenues and commercial return on investment sufficient to develop the technology?  What evidence should be generated?  What are the potential equity consideration of the technology? | Questions as previous box plus:  How usable is the technology?  Should we invest in large scale testing?  What are the barriers/facilitators to adoption?  At what price/performance characteristics are the technology likely to be cost-effective in selected jurisdictions? | Questions as previous boxes plus:  What logistical considerations are required to provide timely access? | Questions as previous boxes plus:  Is the technology likely to be cost-effective in the specific population, position in pathway and jurisdiction at set price?  Should we fund/cover/adopt the new technology?  Should conditions be placed on adoption for restricted coverage, risk-adjusted pricing, and further evidence generation? |

Rosemary Ruiz Seva, Angela Li Sin Tan, Lourdes Marie Sequerra Tejero & Maria Lourdes Dorothy S. Salvacion (2023) Multi-dimensional readiness assessment of medical devices, Theoretical Issues in Ergonomics Science, 24:2, 189-205, https://doi.org/10.1080/1463922X.2022.2064934

## Reconciliation between definition of Early HTA and definitions identified in the literature

| **Authors** | **Terms used** | **Definition** | **Reconciliation with definition suggested** |
| --- | --- | --- | --- |
| Hartz and John, 2008 | Early health economic assessment/early economic evaluation | Economic evaluation in early phases of product development. | Our definition extends beyond economic evaluation into qualitative methods. We avoid use of the term ‘early’ in the definition as it is unclear what early means. |
| Pietzsch and Pate Cornell, 2008 | Early technology assessment | “It is designed mostly to support investment and design decisions at an earlier stage than classic health technology assessment.” | We are consistent with this definition in that we recognise informing innovators about design and other stakeholders (including investors) about value propositions and potential for future market access (thus supporting investment decisions). |
| McAteer, 2011 | Health economics/CEA at the development stage/ early-stage CEA/ supply-side modelling | “Supply side modelling involves estimating the effectiveness of a technology that does not yet exist, let alone one that has undergone head-to-head assessment.” | Our definition extends beyond modelling but picks up the points included in this definition that the technology may not yet exist. We do not mention evidence levels in the headline definition but do set out typical evidence levels in our more detailed definition of the stages of early HTA. |
| Ijzerman and Steuten, 2011 | Early HTA/Very early HTA | “The use of HTA tools and methods to inform biomedical product development and to anticipate further development and market access.” | We used this definition as the basis for ours. We added further detail about audience and timing. We chose to include determining value proposition/s as we consider this a crucial aspect of early HTA. |
| Markiewicz et al, 2014 | Early assessment | “The early examination of the medical, economic, social, and ethical implications of the medical device to determine the potential for incremental value in healthcare.” | Our definition includes the determination of incremental value in healthcare (potential for future market access) but does not explicitly include medical, economic, social and ethical considerations.  We avoid the use of the term ‘early’ and instead say ‘before or alongside the development process’. |
| Rogoswki et al, 2016 | Translational health economics | “The use of theoretical concepts and empirical methods in health economics to bridge the gap between the decision to fund and use a new health technology in clinical practice (the backend of translational medicine) and the decision to invest into its development (the front end of translational medicine).” | Our definition extends beyond health economics including both qualitative and quantitative methods.  We describe timing of evaluation as ‘before or alongside the development process’ whereas Rogowski et al refer to ‘the front end’ and ‘’the back end’ of translational medicine.  We include the aspect of informing a decision to invest in development (the front end) but would argue that the decision to fund and use a new technology is informed by mainstream HTA. |
| Ijzerman et al, 2017 | Early HTA | “All methods used to inform industry and other stakeholders about the potential value of new medical products in development, including methods to quantify and manage uncertainty.” | Our definition is consistent with this definition in referring to a broad range of methods and in including industry and other stakeholders as users of the analysis.  In our definition we situate Early HTA alongside the development of medical products.  Ijzerman et al make the point that early HTA may continue beyond licensing into the early stages of adoption.  In our detailed definition, mainstream HTA is undertaken following the three stages of early HTA when a technology is commercially available. |
| Fasterholdt, 2017 | Early assessment | “Early assessment is different from traditional assessment, by being performed when the initial selection of ideas or rough prototyping has taken place, but prior to large-scale testing or traditional clinical research. Hence, early assessment is based on data from early phases, i.e. feasibility, pilot, or initial effect data generated for the IMT up until Gate N.” | Our definition does not focus only on the period prior to large scale clinical research but includes the whole development process.  We do not refer to evidence available in the main definition, but we do include typical evidence base levels at the different stages in the detailed definition. |
| Bouttell et al, 2019 | Development-focused HTA | “HTA conducted to inform decision making during the development of health technologies” | Our definition is consistent with this.  Bouttell et al only envisaged innovators and investors/funders of health technologies as their audience.  We extend the audience in our definition. |
| Grutters et al, 2019 | Early HTA | Adopts Ijzerman et al, 2017 definition. |  |
| Conrads-Frank et al, 2022 | Early HTA | Adopts Ijzerman et al, 2017 definition. |  |

# References

Bouttell J, Briggs A, Hawkins N. A different animal? Identifying the features of health technology assessment for developers of medical technologies. International Journal of Technology Assessment in Health Care. 2020 Aug;36(4):285-91.

Conrads-Frank A, Schnell-Inderst P, Neusser S, Hallsson LR, Stojkov I, Siebert S, Kühne F, Jahn B, Siebert U, Sroczynski G. Decision-analytic modeling for early health technology assessment of medical devices–a scoping review. GMS German Medical Science. 2022;20.

Fasterholdt I, Krahn M, Kidholm K, Yderstraede KB, Pedersen KM. Review of early assessment models of innovative medical technologies. Health Policy. 2017 Aug 1;121(8):870-9.

Grutters JP, Govers T, Nijboer J, Tummers M, Van Der Wilt GJ, Rovers MM. Problems and promises of health technologies: the role of early health economic modeling. International journal of health policy and management. 2019 Oct;8(10):575.

Hartz S, John J. Contribution of economic evaluation to decision making in early phases of product development: a methodological and empirical review. International Journal of Technology Assessment in Health Care. 2008 Oct;24(4):465-72.

Ijzerman MJ, Steuten LM. Early assessment of medical technologies to inform product development and market access: a review of methods and applications. Applied health economics and health policy. 2011 Sep;9:331-47.

IJzerman MJ, Koffijberg H, Fenwick E, Krahn M. Emerging use of early health technology assessment in medical product development: a scoping review of the literature. Pharmacoeconomics. 2017 Jul;35:727-40.

McAteer H. The use of health economics in the early evaluation of regenerative medicine therapies (Doctoral dissertation, University of Birmingham). 2011.

Markiewicz K, Van Til JA, IJzerman MJ. Medical devices early assessment methods: systematic literature review. International journal of technology assessment in health care. 2014 Apr;30(2):137-46.

Pietzsch JB, Paté-Cornell ME. Early technology assessment of new medical devices. International journal of technology assessment in health care. 2008 Jan;24(1):36-44.

Rogowski W, John J, IJzerman M. Translational health economics. InWorld Scientific Handbook of Global Health Economics and Public Policy: Volume 3: Health System Characteristics and Performance 2016 (pp. 405-440).

## Background questions

Before you provide feedback on the definitions, we would like to know what your background is. Please answer the following questions before proceeding to the actual Delphi survey.

**Please select your age group ***

Please choose **only one** of the following:

- 18 - 29 years old
- 30 - 44 years old
- 45 - 59 years old
- 60+
- Prefer not to say

**Please select to which stakeholder group you belong ***

Please choose **only one** of the following:

- Patient representative
- Policy maker
- Payer
- Industry
- Consultancy
- Provider
- Market authorization
- Academia
- Other

**What type of innovations are you mainly working with? ***

Please choose **all** that apply:

- Medical devices
- Pharmaceuticals
- Service innovations
- Other:

**What type of methods are you most familiar with? ***

Please choose **only one** of the following:

- Quantitative methods
- Qualitative methods
- Both
- Other

**Do you have experience in using or conducting traditional or mainstream HTA? ***

Please choose **only one** of the following:

- No
- Yes, 0-5 years of experience
- Yes, 5-10 years of experience
- Yes, 10-20 years of experience
- Yes, over 20 years of experience

**Do you have experience in conducting early HTA? ***

Please choose **only one** of the following:

- No
- Yes, 0-5 years of experience
- Yes, 5-10 years of experience
- Yes, 10-20 years of experience
- Yes, over 20 years of experience

**Do you have experience in using early HTA, for example in evidence synthesis or to inform decisions? ***

Please choose **only one** of the following:

- No
- Yes, 0-5 years of experience
- Yes, 5-10 years of experience
- Yes, 10-20 years of experience
- Yes, over 20 years of experience

**Do you have experience in using or conducting early dialogue? ***

Please choose **only one** of the following:

- No
- Yes, 0-5 years of experience
- Yes, 5-10 years of experience
- Yes, 10-20 years of experience
- Yes, over 20 years of experience

**Are you willing to participate in a second round of this process? If so, please provide your e-mail address below. If you do not provide your e-mail address, you will not be invited to participate in the next round.**

Please write your answer here:

We will use this only to send a personal link to the next round(s) of the Delphi process. Your answers will be handled anonymously.

## Delphi Exercise

Below, you will first find a question on the terms used, followed by the proposed definitions of early HTA, development-focused HTA and early dialogue. Below each definition you will find a free text box where you can state if you agree with the definition and if you have any suggestions for rewording, additions or other changes. Any feedback is greatly appreciated. In the final text box you can make other more general comments, for example on the exercise, your experience or any literature that might be relevant.

**Do you agree to use the terms early HTA, development-focused HTA and early dialogue? Why (not)? If you do not agree, please provide alternative terms. ***

Please write your answer here:

**Early HTA is a formal, systematic, transparent and multidisciplinary process that uses explicit methods, both quantitative and qualitative, to explore the potential and/or expected value of a health technology*, including the associated uncertainty, before or alongside the technology development process. Stages at which early HTA can be undertaken include the concept/discovery stage, prototype/proof of concept stage and research/evidence development stage. The stages impact upon the evidence/data available, the questions to be answered, methods to be used and the audience for the work.**

**Do you agree with this aspect of the definition? What suggestions do you have? ***

Please write your answer here:

**The purpose is to provide innovators with insight about the potential value for the health system and commercial viability of a technology, and to inform decision-making about the (clinical) need, design of a technology, positioning of the technology in the care pathway, further research needed to prove value and potential for future market access and adoption, in order to promote a high-quality health system.**

**Do you agree with this aspect of the definition? What suggestions do you have?***

Please write your answer here:

**Development-focused HTA is a specific type of early HTA that is mainly undertaken in the first two stages of early HTA (concept/discovery stage and prototype/proof of concept stage).  Its purpose is to provide innovators with insight about the potential (commercial) viability and design of a technology and the potential for future market access. It may also provide information for decision-makers about the potential of a technology in development to contribute to a high-quality health system.**

**Do you agree with this definition? What suggestions do you have?***

Please write your answer here:

**Early dialogue is the exchange between manufacturers and public institutions to obtain guidance on the evidence requirements for regulatory and reimbursement purposes. The purpose is to facilitate the flow of information between manufacturers, competent authorities (CA), and HTA agencies. Also known as early advice or (early) scientific advice.**

**Do you agree with this definition? What suggestions do you have?***

Please write your answer here:

**The table in Section 4 includes a detailed description of the stages of early HTA in comparison with mainstream HTA.**

|  | **Early HTA** | | | **Mainstream HTA**  **Implementation stage** |
| --- | --- | --- | --- | --- |
|  | **Stage 1 – concept/discovery stage** | **Stage 2 – prototype/proof of concept stage** | **Stage 3 – research and evidence generation stage** |  |
| **Technology Readiness Levels (TRL)**  **(Based on Seva et al, 2023)** | Up to and including TRL 4 from needs assessment, concept, development of a working prototype, including the observation and reporting of basic principles, development of an early prototype, bench and animal testing.  Proof of concept established. | TRL 5-6 – small scale pilot and feasibility testing. | TRL 7-8 – large-scale testing leading to regulatory approval. | TRL 9 – market access, adoption and post-market surveillance. |
| **Typical technology-specific evidence level** | No effectiveness evidence specific to the technology available.  Available technology-specific evidence may include:  Bench, in silico and animal studies, formulation, pharmacokinetic, absorption, distribution, metabolism and excretion (ADME) studies.  User studies of an early prototype. | Evidence of safety and effectiveness from small sample.  Usability/patient acceptance studies.  Pre-clinical studies, including Good Laboratory Practice, animal safety and toxicity.  Phase 1 and Phase 2a clinical trials conducted. | Effectiveness study.  May be randomised controlled trial or observational evidence depending on regulatory requirements.  Phase 2b and Phase 3 clinical trials conducted. | Regulatory evidence base available.  Evidence of clinical effectiveness, quality of life and cost implications (clinical utility) may be limited to certain settings or populations.  Post-market / Real-World studies. |
| **Typical project vignette** | **Technology-driven** - either an emerging and generalised technology with broad application across several potential indications, or a technology with specific features requiring a target indication, setting and position in a pathway.  **Needs-driven** - no technology yet specified, with emphasis on identifying and designing features required to realise a patient, payer or innovator improvement. | Potential indication and/or features have been narrowed down.  Position in pathway and setting may still be unclear. | Product and market development may be continuing.  Large scale testing.  Indication, position in pathway and setting should be clear. | Indication is clear from regulation. |
| **Audience** | Innovators/investors (industry/academic/clinician)  Funders or insurers  Technology transfer offices  Research funders  Patients and their advocate groups | Innovators/investors  Funder or insurer  Technology transfer offices  Health service users  Patients and their advocate groups | Innovators/investors  Funder or insurer  Technology transfer offices  Health service users  Patients and their advocate groups | Funders or insurers  Health service users  Patients and their advocate groups  Technology transfer offices |
| **Example questions to be answered** | What is/are the current pathway/s?  How would the technology change the care pathway?  What is the room for improvement?  What other technologies are on the horizon, which may change the competitive, therapeutic environment?  Do the potential or target claims/value proposition impact health, cost, access, equity, location of care, efficiency, sustainability?  What characteristics does the technology need to deliver on these claims?  What evidence is required to demonstrate that the technology is likely to deliver value as defined by the chosen decision-maker/s?  How does the decision maker weight the different elements of impact?  What is the minimum level of outcomes the expected costs could support, given threshold prices?  What is the maximum costs the expected outcomes could support, given threshold prices? Are the expected revenues and commercial return on investment sufficient to develop the technology?  What evidence should be generated?  What are the potential equity consideration of the technology? | Questions as previous box plus:  How usable is the technology?  Should we invest in large scale testing?  What are the barriers/facilitators to adoption?  At what price/performance characteristics are the technology likely to be cost-effective in selected jurisdictions? | Questions as previous boxes plus:  What logistical considerations are required to provide timely access? | Questions as previous boxes plus:  Is the technology likely to be cost-effective in the specific population, position in pathway and jurisdiction at set price?  Should we fund/cover/adopt the new technology?  Should conditions be placed on adoption for restricted coverage, risk-adjusted pricing, and further evidence generation? |

**Do you agree with this detailed description? What suggestions do you have?***

Please write your answer here:

**Do you have any other suggestions or remarks you would like to share with the Working Group?**

Please write your answer here:

**Thank you very much for sharing your feedback. By clicking 'submit' you will submit your results and can no longer make changes.**

Thank you for participating in this Delphi exercise, your input is greatly appreciated. We will now take into account all feedback and alter the definitions accordingly. We expect to send you a new Delphi exercise in January 2024. We hope you will also participate in this second round. Thanks again!

# SM3 – Second round Delphi survey

Thank you for your helpful feedback to the first round of our Delphi exercise. As you may recall, in this study we aim to develop a clear definition of the term “early HTA”. Such a definition is currently lacking and there is confusion with related terms such as “early dialogue” and “development-focused HTA”. Reaching consensus on what is meant by early HTA will form a foundation for discussion and dialogue about the best methods to adopt and improve the profile of the field. You are part of a panel of global experts who will be taking part in this study and influencing the output of this research. Because you provided your email address in the first survey, you are invited to take part in this second survey.

This exercise is expected to take no more than 20 minutes of your time. Once you click 'Next', you will be directed to the feedback page, where we briefly discuss what feedback we received in the first round and how we used this feedback to make changes. Next, we will present the revised definition, followed by a background questionnaire, and, finally, the Delphi exercise. At the end of the survey you will be asked if you consent to being acknowledged in the peer-reviewed paper that describes the Delphi study and result.

Please note that you must submit the questionnaire for us to see your answers. Your responses will be handled anonymously. If you do not wish to take part in this second round of the Delphi process, please send an email to Janneke Grutters (janneke.grutters@radboudumc.nl) or Janet Bouttell (Janet.Bouttell@nuh.nhs.uk). We will then remove your email from the list of participants and make sure that you do not receive any reminders.

The deadline for submitting your answers is February 29, 2024. If you have any questions regarding this Delphi survey, you can contact Janet or Janneke, at any point in the process.

There are 13 questions in this survey.

## Feedback and changes made

In total, 132 people responded to the first round of this Delphi exercise. Out of these 132, 119 respondents provided their email addresses and are invited to this second survey.

Overall, there was considerable agreement on the terminology and definitions we proposed. Some respondents suggested alternative terms. Below we will focus on the three terms we used in the first round and how we will continue with these.

**Development-focused HTA**

Multiple responses regarded the relation between early HTA and development-focused HTA, and whether it was necessary to distinguish them. And, if development-focused HTA is a subset of early, should we not include other subsets? Because of these comments, we decided to focus on one term, being ‘early HTA’, and drop development-focused HTA. In the paper that will accompany the new definition of early HTA, we will discuss how early HTA relates to other terms such as development-focused HTA, but we will not continue with this term in developing a consensus definition.

**Early dialogue**

Multiple suggestions were provided to add different stakeholders to the definition of early dialogue. However, early dialogue was deliberately defined to specifically describe the dialogue between regulators, HTA agencies and innovators. Since there are currently multiple initiatives ongoing, such as the new European HTA regulation, that will impact the terms and definition associated with early dialogue and/or early scientific advice, we concluded that it is at this moment too early to develop a consensus definition. As a result, also the term ‘early dialogue’ was dropped from this survey. In the paper that will accompany the new definition of early HTA, we will discuss the role of early dialogue and its relationship to early HTA.

**Early HTA**

Although other terms were suggested by some respondents, most respondents agreed to use the term ‘early HTA’. The initial definition was based on the definition of HTA that is currently in the HTA glossary. Since many suggestions were provided on those parts of the definition that were based on the HTA definition, we decided to separate them. In addition, many suggestions were provided to shorten the definition. A number of survey participants questioned whether early HTA has the aim to ‘promote a high quality, equitable and efficient health system’. We believe that early HTA does have that aim as it anticipates the assessment of value which will be made later in mainstream HTA. Early HTA may also extend this assessment to look at return on investment for the developer. If only return on investment is examined, from an innovator perspective, without exploring the health system consequences, this would not be regarded as early HTA, but for example as a business case. Related to this, we received comments relating to whether early HTA is always formal, systematic and transparent. Although we do believe that early HTA should ideally be formal, systematic and transparent, we recognise that this is not always the case. We therefore changed this into ‘explicit’.  Another suggestion was to emphasize the role of early HTA in evaluating the potential value of a technology before it is actually being developed, in the idea phase. We therefore included that the technology can be an actual or a conceptual health technology. 
We also talked to the Chair of the English Editorial Board of the HTA Glossary, to understand what was needed for the definition of early HTA to be included in the HTA glossary. Based on this, the structure of the definition was slightly changed. The first phrase of the definition has to be the category that the new definition will come under. In our case, the first phrase will be ‘health technology assessment’ that is already in the glossary. We also added two notes for clarification: the health technology definition that is already in the glossary, and one on what we mean by value. This second one is based on the similar note in the HTA definition, but we added ‘for example’ to allow for a broader range of stakeholders.

Following this conversation and the feedback from the first round of the Delphi, a new definition was proposed to and discussed in the Working Group. Following this discussion, a final definition was proposed by the Working Group, which can be found on the next page. In addition, we received a lot of very helpful feedback to the Table. We will update the Table based on this feedback, and will include it in the paper that we will write on this study. Since we do not aim to reach consensus on this Table, and we want to minimize the burden of completing the survey, we left it out of this second round.

If you are interested to receive a more specific response to how your individual feedback was incorporated, please send us an email: janneke.grutters@radboudumc.nl or [Janet.Bouttell@nuh.nhs.uk](mailto:Janet.Bouttell@nuh.nhs.uk).

## Proposed definition of early HTA

**Early health technology assessment; early HTA:**

A health technology assessment conducted to inform decisions about subsequent development, research and/or investment by explicitly evaluating the potential value^1^ of a conceptual or actual health technology^2^.

^1^ The dimensions of value for a health technology may be evaluated by examining the intended and unintended consequences of using a health technology compared to existing alternatives. These dimensions often include clinical effectiveness, safety, costs and economic implications, ethical, social, cultural and legal issues, organisational and environmental aspects, as well as wider implications, for example for the patient, relatives, caregivers, innovator and the population. The overall value may vary depending on the perspective taken, the stakeholders involved, and the decision context.

^2^ An intervention developed to prevent, diagnose or treat medical conditions; promote health; provide rehabilitation; or organize healthcare delivery. The intervention can be a test, device, medicine, vaccine, procedure, program or system.

## Background questions

**In which region are you based at the moment? ***

Please choose **only one** of the following:

- Asia
- Africa
- Australia
- North America
- South America
- Europe
- Other

**Which categories of countries are the focus of your work? ***

Please choose **all** that apply:

- Low income countries
- Middle income countries
- High income countries

**Do you conduct 'standard' HTA to inform reimbursement, adoption or similar decisions? ***

Please choose **all** that apply:

- Yes, on behalf of HTA agencies
- Yes, on behalf of industry
- Yes, on behalf of healthcare institutions (e.g. hospitals)
- Yes, on behalf of national funding bodies
- No
- Other:

**Do you conduct early HTA? ***

Please choose **all** that apply:

- Yes, on behalf of HTA agencies
- Yes, on behalf of industry
- Yes, on behalf of healthcare institutions (e.g. hospitals)
- Yes, on behalf of national funding bodies
- No

**Other:**

Do you want to share any further information on your background that might be relevant for this survey?

Please write your answer here:

## Delphi exercise

Below, you will find the proposed definition of early HTA. Below the definition you will find a Likert scale ranging from ‘strongly agree’ to ‘strongly disagree’. Consensus will be considered to be reached if 70% of respondents agree or strongly agree on the proposed definition. If consensus is not reached, a third round may be considered. Below the Likert scale, you will find a free text box where you can provide suggestions for rewording, additions or other changes. Any feedback is greatly appreciated. In the final text box you can make other more general comments, for example literature that might be relevant for the paper, feedback on the exercise, or your personal experience.

**Early health technology assessment; early HTA:**

A health technology assessment conducted to inform decisions about subsequent development, research and/or investment by explicitly evaluating the potential value^1^ of a conceptual or actual health technology^2^.

^1^ The dimensions of value for a health technology may be evaluated by examining the intended and unintended consequences of using a health technology compared to existing alternatives. These dimensions often include clinical effectiveness, safety, costs and economic implications, ethical, social, cultural and legal issues, organisational and environmental aspects, as well as wider implications, for example for the patient, relatives, caregivers, innovator and the population. The overall value may vary depending on the perspective taken, the stakeholders involved, and the decision context.

^2^ An intervention developed to prevent, diagnose or treat medical conditions; promote health; provide rehabilitation; or organize healthcare delivery. The intervention can be a test, device, medicine, vaccine, procedure, program or system.

**Do you agree with this definition? ***

Please choose **only one** of the following:

- Strongly agree
- Agree
- Neither agree nor disagree
- Disagree
- Strongly disagree

**Do you have any suggestions on the definition?**

Please write your answer here:

**Do you have any other suggestions or remarks you would like to share with the Working Group?**

Please write your answer here:

**Follow-up**

You input is highly appreciated. When we report on the Delphi process and results, we would like to acknowledge your input by mentioning you in the Acknowledgements section of the manuscript. If you prefer not to be acknowledged, you will be included in the total of anonymous respondents. Whatever you choose, your responses will be handled anonymously.

**Do you consent to being acknowledged in the peer-reviewed paper that describes the Delphi study and results? ***

Please choose **only one** of the following:

- Yes
- No

**If yes, please state your name, title and affiliation as you want us to list them in the paper.**

Please write your answer here:

**Thank you for providing your feedback. By clicking 'submit' you will submit your results and can no longer make changes.**

Thank you for participating in this Delphi exercise, your input is greatly appreciated. We will now take into account all feedback. Your results will be sent to the Working Group in an anonymous form. Thanks again!

# SM4 – Information sheet for participants

**Developing consensus on definitions of early HTA, development-focused HTA and early dialogue – information sheet**

Dear colleague,

You are invited to participate in our Delphi study. In this study we aim to develop a clear definition of the term “early Health Technology Assessment (early HTA)”.

Recently, an international joint task group of the International Network of Agencies for Health Technology Assessment (INAHTA) and Health Technology Assessment International (HTAi) developed a new definition of HTA. They define HTA as “… a multidisciplinary process that uses explicit methods to determine the value of a health technology at different points in its lifecycle. The purpose is to inform decision-making in order to promote an equitable, efficient, and high-quality health system”.^1^

While HTA is typically thought of as a process associated with pharmaceuticals and devices, the joint task group have also noted that a health technology is any intervention developed to prevent, diagnose, or treat medical conditions; promote health; provide rehabilitation; or organize healthcare delivery. The intervention can be a test, device, medicine, vaccine, procedure, program, or system.

HTA is increasingly used in the earliest phases of development of a health technology, from the idea phase up, through to market approval and as part of ongoing pricing and regulatory negotiations. Developments such as the new EU Regulation on HTA^2^ are expected to further increase the relevance of HTA in these early stages.

However, multiple terms are currently being used to describe early HTA activities. Moreover, it is unclear how terms such as early HTA, development-focused HTA and early dialogue or early (scientific) advice relate to each other. Reaching consensus on what is meant by each of these terms will form a foundation for discussion and dialogue about the best methods to adopt and improve the profile of the field. The study is conducted and coordinated by the Health Technology Assessment international (HTAi) Working Group on early HTA, led by Janet Bouttell (Nottingham University Hospitals Trust, UK) and Janneke Grutters (Radboudumc, the Netherlands).

If you agree to participate in the Delphi study, you are part of a cohort of global experts who will be taking part in this study and influencing the output of this research. We ask you to take part in two to three rounds of web-based surveys. In the first round, you will be provided a provisional definition of the terms “early HTA”, “early dialogue” and “development-focused HTA”, including related terms that were found in a literature search, as provided by the Working Group. You are asked to qualitatively comment on these definitions. Any feedback is greatly appreciated. All responses will be anonymously shared and discussed with the Working Group, who will adapt the definitions based on your feedback. In the second round you will be provided with the altered definitions and asked to either agree or disagree on a Likert rating scale (strongly agree, agree, neither agree nor disagree, disagree, strongly disagree). You will also be asked to provide explanations and/or recommendations. Consensus will be considered to be reached if 70% of respondents agree or strongly agree. If consensus is not reached on a particular aspect a third round may be considered.

Each survey round will be online for up to 4 weeks and reminder emails will be sent approximately 14 days after the initial invitation. Only those who complete round 1 will be invited to participate in the next rounds. For this you will be asked to provide your e-mail address in the first survey, so that we can personally invite you for the second round. Your email address will only be used for the invitation for the second round; your responses will be handled anonymously.

Participating in this online survey is voluntary, and you can withdraw from the study at any time by replying to the invitation email. We expect each round to take no more than 30 minutes of your time. Your data will be used in an anonymized format and your name will not be used in any reports or publications resulting from the study, unless you explicitly consent to this. In the second round of the Delphi you will be asked if you consent to being acknowledged in the peer-reviewed paper that describes the Delphi study and results.

The Delphi study abides by the ethical requirements of the Radboud university medical center, aiming to assure responsibility in the conduct of the research project. A copy of the ethics review approval is available on request. All participants will be asked to complete a consent form at the beginning of the web-Delphi process.

If you have any questions regarding this Delphi survey, you can contact Janet Bouttell (Janet.Bouttell@nuh.nhs.uk) or Janneke Grutters (janneke.grutters@radboudumc.nl), at any point in the process.

1 Definition of HTA in the HTA glossary. http://htaglossary.net/health+technology+assessment

2 Regulation (EU) 2012/2282 on Health technology assessment (HTAR). https://health.ec.europa.eu/health-technology-assessment/regulation-health-technology-assessment_en

# SM5 – Protocol

**DELPHI STUDY PROTOCOL – Developing consensus on definitions of early HTA, development-focused HTA and early dialogue**

**BACKGROUND**

Early HTA is a broad church, encompassing work to inform developers during the development of a technology as well as users/payers/reimbursement agencies in the early stages of adoption, particularly during arrangements where coverage is conditional on further research or where evidence is scarce Often authors do not explicitly state why they consider their study to fall within the remit of early HTA. Early HTA is increasingly used, and has great potential in reducing research waste, ensuring that investment goes to technologies which can make a difference and that technologies in development are optimized to ensure they are fit for purpose.

Early HTA methods such as health economic modelling and stakeholder engagement can be used to explore the potential value, usefulness and required characteristics of a technology in development using different scenarios based on real-world settings. This is important as different characteristics may be required depending on where in a clinical pathway a technology will be used. In low and middle income countries (LMIC) early HTA may be of particular use in specifying the technologies which would have the most impact, were they to receive investment.

There is considerable debate over methods of early HTA. For health economic modelling there are some who argue that methods should always meet standards set in reimbursement agencies’ reference cases. Others favour simpler approaches earlier in development, for example, using scenario analysis and one way sensitivity analysis to deal with uncertainty. The literature often offers little guidance in which approach to take as much early modelling remains internal due to commercial sensitivity. Studies that are reported often lack key information such as the stage of development of the technology, the purpose of the modelling and the limitations in the evidence base. Similarly for methods of stakeholder engagement, there is a balance to be struck between the complexity and scale of studies and the resources available to the decision maker.

Clear guidance on terminology, methods and reporting taking into account the heterogeneity in the field would greatly assist practitioners in both industry, academia, consultancy and decision-making bodies, as well as journals seeking to ensure the quality of published work. As a first step, a Working Group is initiated under the Health technology Assessment international (HTAi) society to reach consensus on a definition of “Early HTA” and the related terms “Development-focused HTA” and “Early Dialogue”.

**METHODS**

The Delphi method is a practical and structured method of obtaining opinions on a given question from a range of experts and can be used to gain consensus among a group of experts or informed respondents that constitute the Delphi panel. The respondents will take part anonymously in sequential questionnaires that constitute different rounds and each round is refined based on feedback from the previous version. After each round, the group responses will be fed back to the Delphi panel who can reconsider their views based on this report of the group views.

*Selection of participants*

We will obtain views from individuals with varied professional roles, perspectives and experiences in the application or use of (early) Health Technology Assessment. Participants will be invited personally within the professional networks of the Working Group members. In addition, the survey will be advertised through social media and the HTAi society.

*Survey design and execution*

First, a rapid review will be performed to obtain a list of terms and definitions currently used. The results of this rapid review will be shared and discussed with the members of the Working Group (WG). The WG consists of a group of 15-20 global experts in the field, from different professional backgrounds. The WG will propose definitions for “early HTA”, “development-focused HTA” and “early dialogue”. In the first round of the Delphi process, these definitions, including the background information, are shared with the participants. The purpose of this round is to identify in a qualitative way the ideas and feedback on these definitions. This round will provide qualitative information about stakeholder views on possible improvements. In addition, some questions on characteristics (e.g. age, professional background, experience) of respondents are included.

The results will be shared anonymously with the WG and discussed. Based on these discussions, the definitions are altered. A summary of the responses will be fed back to all participants for further rounds of questions. The second round will quantify the degree of consensus on the altered definitions. Round 2 makes use of statements to which participants can either agree or disagree on a Likert rating scale (strongly agree, agree, neither agree nor disagree, disagree, strongly disagree). They will also be asked to provide explanations and/or recommendations. Consensus will be considered to be reached if 70% of respondents agree or strongly agree. If consensus is not reached on a particular aspect a third round may be considered.

We will use Castor software to develop the online survey. Each survey round will be online for up to 4 weeks and reminder emails will be sent approximately 14 days after the initial invitation. Only those who complete round 1 will be invited to participate in the next rounds.

**Reporting**

Results of the rapid review, WG discussions and Delphi process will be reported in a scientific article. In addition, the final definitions will be proposed for inclusion in the HTA Glossary.

# SM6 – Working group’s response to round 1 feedback

Below we explain the working group responses to feedback received in the first round and how that was taken into account in the different aspects of the definition. We will focus on six aspects of particular importance that were considered by the working group in revising the definition.

**Promotion of a high quality health system**

In the definition circulated in the first round of the survey we included the phrase ‘in order to promote a high quality health system’. Many comments from panellists questioned this, given that this work is often undertaken for or on behalf of industry with a view to generating profits for the commercial enterprise. Our working group strongly felt that, before the commercial potential of a technology can be explored, its impact on the patient and the healthcare system, including its impacts on costs, health outcomes, efficiency and equity must be assessed. This assessment will be from a health system perspective where the payer is a health service, rather than an individual patient. A subsequent commercial business case may be based on this assessment. When the payer is an individual patient, it is the benefit of the technology to that patient which forms the basis of the value proposition and commercial business case.

**Systematic, transparent, and formal**

A number of panellists questioned whether early HTA could be said to be ‘systematic, transparent and formal’. After detailed discussion the working group considered that good practice in early HTA required the application of established methods suitable for the specific purpose of the analysis that were disclosed to the audience for whom the work was undertaken. The working group was satisfied that such best practice early HTA would align with these terms.

**Definition of technology**

In our first definition we included the existing HTA glossary definition of a health technology. This wide definition recognises that early HTA work is undertaken across the spectrum of health technologies with work on pharmaceuticals and devices but also work on healthcare delivery systems and processes. Panellists suggested that we add software to the definition of technology. The working group considered that software and digital health technologies were a sub-set of devices, which are included in the definition, and therefore decided to keep the definition of technology the same as the HTA glossary for the second round of the Delphi process.

**Definition of value**

In our first round survey, we defined value exactly as the HTA Glossary definition of health technology assessment. Many panellists questioned whether value was different when early HTA is used to inform (commercial) innovators and investors during the development of a health technology. The working group considered that the ability of a technology to generate value for an innovator (or by extension an investor) was dependent on it first generating value for the health system and/or the patient as well as the extent to which the innovator is able to capture some, or all of the value generated.

In the second definition, we added innovator to the list of stakeholders for whom the technology may have wider implications. We also included in the second definition that ‘the purpose of early HTA was to inform decisions about subsequent development, research and/or investment’. The working group felt that these changes sufficiently captured the extent to which value may have a broader meaning for commercial innovators and investors.

**Stakeholders**

In our first definition we included ‘the patient, relatives, caregivers and the population’ as those stakeholders who would feel the wider implications of a health technology. Panellists to the first round stressed that early HTA should have a focus on a collaborative, interdisciplinary approach and include reference to co-design involving patients and clinical experts. The working group felt that definition of early HTA as a subset of HTA implicitly included the ‘multidisciplinary process’ element of the HTA definition. As discussed above, innovators were added to the list of stakeholders in order to explicitly recognise their interest in the output from early HTA, although the working group did note that their interests were already included in the final sentence of the definition of value; ‘the overall value may vary depending on the perspective taken, the stakeholders involved and, the decision context.’

**Stage of development**

Many panellists commented on the difficulty in determining the stages of development where early HTA is undertaken and the point at which HTA is no longer ‘early’. In the first definition we specified that early HTA could be used at three stages: ‘concept/discovery’, ‘prototype/proof of concept’ and ‘research/evidence development stage’. These stages were selected with care to ensure that they were applicable to all kinds of technologies including system and process innovation. Many panellists felt that we were leaving the cut-off between early and ‘not early’ HTA too late. The survey materials for the first round included a detailed table which split early HTA into the three stages mentioned above. On reflection, the working group acknowledged that in the third research/evidence development stage, the purpose of HTA may be more focused on meeting the needs of a payer or reimbursement agency. As such, Stage 3 may be a stage where early HTA merges, and overlaps, with other forms of HTA. In order to reflect this development, we removed the three delineated stages from the second definition to instead focus on the purpose of the evaluation, namely ‘to inform decisions about subsequent development’.

Some panellists questioned whether early HTA could be conducted before the technology development process. The working group were keen to retain this aspect of the definition given the promise of early HTA in needs-led innovation to evaluate whether an idea is worth pursuing. In the second definition we include the phrase ‘conceptual or actual health technology’ to reflect the fact that, while the evidence may be lacking and/or highly uncertain, early HTA can be undertaken before there is any prototype or proof of concept.

# SM7 – Panellists completing both rounds one and two who agreed to be acknowledged

Lucia Reis do Nascimento, Nurse, Health Technology Assessment Nucleus (HTEN), University Hospital of the Federal University of Pernambuco

Leon Bijlmakers, PhD, IQ Health science department, Radboudumc

Alessio Cortiana, PhD Candidate, School of Medicine and Surgery, University of Milan Bicocca

Zareen Khan, PhD Candidate - Health Economics, Norwegian University of Science and Technology

Yot Teerawattananon, Co-director of the Medical Innovation Development and Assessment Support (MIDAS), Thailand

Abhirup Dutta Majumdar, Senior Statistician (HEOR & RWE), PharmaQuant Insights, India

Mr. Christopher Munoz, Philippine Alliance of Patient Organizations

Linn Nathalie Støme, Researcher, Oslo University Hospital

Dr. Melinda Goodall, Goodall HTA Consulting Ltd

Katherine Payne, Professor of Health Economics, The University of Manchester

Chris van Lieshout, MSc, Department of Epidemiology and Health Economics, Julius Center for Health Sciences and Primary Care, University Medical Center Utrecht, Utrecht University

Miranda L. van Hooff, Department of Orthopedic Surgery, Radboud University Medical Center, Nijmegen, The Netherlands; Department of Research, Sint Maartenskliniek, Nijmegen, The Netherlands

Erika Maria Henriques Monteiro, Head of the Technological Innovation Management Unit in Health, University Hospital of UFJF, Ebserh branch.

Rashmi Joglekar, HEPR Strategy Manager, Medtronic Australasia

Marisa Santos, MD, PhD, Instituto Nacional de Cardiologia, Brazil

Renee Else Michels, MSc, Erasmus School of Health Policy and Management

Victoria Hurtado, MSc, Ministry of Health, Chile; Universidad de Santiago de Chile (USACH)

Dr. Eddy Adang, Associate Professor Health Economics, Radboudumc, Nijmegen, Netherlands

Manuel Donato, Pham, MSc, National Commission for the Evaluation of Health Technologies and Clinical Excellence (CONETEC), Ministry of Health of Argentina

Ryagina Veronika, Senior Expert, Department of Methodological Support for Comprehensive Assessment of Technologies in Healthcare, Center for Healthcare Quality Assessment and Control

Denny John, Professor, Faculty of Life and Allied Health Sciences, MS Ramaiah University of Applied Sciences, Bengaluru, India

Prof. Dr. Manuela A. Joore, Maastricht UMC+ & CAPHRI, Maastricht University, The Netherlands.

Xavier G.L.V. Pouwels, Assistant Professor in Health Technology Assessment, Health Technology & Services Research Section, TechMed Centre, Faculty of Behavioural, Management & Social Sciences, University of Twente, Enschede, The Netherlands

Dr. Hanin Farhana Kamaruzaman, Senior Principal Assistant Director, Malaysian Health Technology Assessment Section (MaHTAS)

Yanga Nokhepheyi, Researcher Mediclinic Group

Prof. Dr. Veerle MH Coupé, Department of Epidemiology and Data Science, AmsterdamUMC, The Netherlands

Dr. Tom Kenny, Patient Advocacy Lead Rare Diseases, Chiesi

Valerie J. King, MD, MPH, Professor and Research Director, Center for Evidence-based Policy, Oregon Health & Science University, Portland, Oregon, USA

Eon Ting, Director Value & Access, AstraZeneca, Canada

Veronica Gallegos, PhD, CENETEC, México

Frédérique Debroucker, Senior Director, Health Economics Policy and Reimbursement, Western Europe, Medtronic

Jaime Kristoffer Punzalan, Physician, School of Medicine, Ateneo de Zamboanga University

Dan Swain, Principal Consultant, Dan Swain Economics

David Hailey, Professorial Fellow, University of Wollongong

Tan Nguyen, Casual Research Fellow, Deakin Health Economics, Institute for Health Transformation, Deakin University

Megan Bohensky, Head of Market Access, Asia-Pacific, BeiGene

Geneviève Plamondon, MSc, Scientific Advisor, Institut national d'excellence en santé et services sociaux (INESSS)

Mirre Scholte, PhD, Department of Clinical Epidemiology and Medical Technology Assessment, Maastricht University Medical Center, Maastricht, the Netherlands

Professor Tracy Merlin, Director, Adelaide Health Technology Assessment (AHTA); Head, School of Public Health, University of Adelaide

Ewan Gray, Associate Director, HEOR, Grail

Monica Ferrie, Chief Executive, Genetic Support Network Victoria

Simon Verdonck, Market Access Senior Consultant Europe & Asia-Pacific, Alira Health

Karin Wilbe Ramsay, PhD, Swedish Agency for Health Technology Assessment and Assessment of Social Services

Toby Gould, Director, HTAccess Consulting

Kimberley-Jane Crawford, Volunteer Board Director, Asbestosis and Mesothelioma Association of Australia Limited (AMAA)

Dr. Pooyeh Graili, Principal, Quality HTA; Adjunct lecturer, University of Toronto; Visiting scholar, Toronto Metropolitan University

Sarah Norris, Associate Professor of Practice, HTA, University of Sydney, Australia

Mark Brand, Principle Consultant, BRANDTECH Health Technology Consulting, Western Cape, South Africa

Lesley Dunfield, Senior Advisor Partnerships, CADTH

Borja García-Lorenzo, PhD, Biosistemak Institute

Dr. Nicola McMeekin, HEHTA, School of Health and Wellbeing, University of Glasgow

Ms. Alicia Norman, Macquarie University Centre for the Health Economy, Sydney, Australia.

Rachael Anderson, Market Access Director, Australia and New Zealand, AstraZeneca Pty Ltd

Iain Tatt, PhD, F. Hoffmann-La Roche Ltd

Joost J. Enzing, PhD, Erasmus School of Health Policy & Management, Erasmus University Rotterdam, Rotterdam, The Netherlands; Zorginstituut Nederland, Diemen, The Netherlands

Dr. Paul Fennessy, Principal, Paul Fennessy Advisory

Associate Professor Bonny Parkinson, Macquarie University Centre for the Health Economy, Macquarie University, Sydney, Australia

A.D.I. van Asselt, PhD, University of Groningen, University Medical Center Groningen

Sabine E Grimm, PhD, Maastricht University Medical Center+, Netherlands

Anirban Basu, Professor of Health Economics, Stergachis Family Endowed Director, The CHOICE Institute, University of Washington, Seattle

Marcia Tummers, IQ Health Science Department, Radboud University Medical Center, Nijmegen, The Netherlands.

Joan Fibla-Reixachs, MSc, HTA Scientist, Evaluation of Innovation and New Technologies Unit, Research and Innovation Directorate, Hospital Clínic Barcelona

Dr. Kavita Kachroo, Chief Operating Officer, Scientist-E, Kalam Institute of Health Technology, India

Prof. em. Albert Boer, MD, PhD, ESHPM, Erasmus University Rotterdam

Marina Richardson, Associate Director, HTA Methods and Health Economics, ICER

Jill Berensen, Dragon Claw Charity Ltd.

Liesl Strachan, Director, Global Health Policy, Health Economics & Reimbursement

Iben Fasterholdt, Senior Researcher and Research Fellow, CIMT – Centre for Innovative Medical Technology, Odense University Hospital, Odense, Denmark; Toronto General Hospital Research Institute, University Health Network, Toronto, Canada

Maria Pinelli, PhD Student, Department of Management, Economics and Industrial Engineering, Politecnico di Milano

Aline Topouchian, PhD, Senior Manager, Market Access THV - EMEA-Canada-Latin America, Edwards Lifesciences

Pablo Lázaro de Mercado, MD, PhD, MBA, Independent Health Services Researcher

Silke Anna Theresa Weber, MD, PhD, Botucatu Medical School, FMB-UNESP, Botucatu-SP, Brazil; NATS Hospital das Clínicas HCFMB, Botucatu-SP, Brazil

Cecily Gilbert, Ms, Research Specialist in Digital Health Information, Centre for Digital Transformation of Health, The University of Melbourne

Robin Vermeulen, Department of Medical Imaging, Radboud University Medical Centre, Nijmegen, The Netherlands

Dr. Sara Graziadio, PhD, York Health Economics Consortium

Ties Hoomans, PhD, Care Policy and Evaluation Centre, London School of Economics and Political Science

Manuel Antonio Espinoza, MD, MSc, PhD, Associate Professor, Faculty of Medicine, Pontificia Universidad Católica de Chile; Principal Investigator, Center for Cancer Prevention and Control, Chile; Chair, HTAi Latam Policy Forum

Gabriela Restovic, Associate Director of Market Access, Novocure, Spain
